# Semantic Resources for Managing Knowledge in Battery Research

**DOI:** 10.1002/cssc.202500458

**Published:** 2025-07-15

**Authors:** Simon Clark, Corsin Battaglia, Ivano E. Castelli, Eibar Flores, Lukas Gold, Christian Punckt, Simon Stier, Philipp Veit

**Affiliations:** ^1^ SINTEF Industry Battery Technology Strindvegen 4 Trondheim 7034 Norway; ^2^ Empa ‐ Swiss Federal Laboratories for Materials Science and Technology Ueberlandstrasse 129 Dübendorf 8600 Switzerland; ^3^ Department of Information Technology and Electrical Engineering ETH Zurich Gloriastrasse 35 Zurich 8092 Switzerland; ^4^ EPFL School of Engineering, Institute of Materials Station 15 Lausanne 1015 Switzerland; ^5^ Department of Energy Conversion and Storage Technical University of Denmark Agnes Nielsens Vej 301 Kgs. Lyngby DK‐2800 Denmark; ^6^ Department Digital Transformation Fraunhofer Institute for Silicate Research ISC Neunerplatz 2 97082 Würzburg Germany; ^7^ Helmholtz Institute Ulm for Electrochemical Energy Storage (HIU) Karlruhe Institute of Technology D‐89081 Ulm Germany

**Keywords:** batteries, knowledge bases, linked data, ontology, semantic technologies

## Abstract

Semantic technology is revolutionizing how the battery research community collaborates. It is becoming even more important as artificial intelligence agents emerge in the field. This article explores the role of semantic resources in advancing battery research by enabling the formalization of knowledge in a way that can be understood by both people and computers. Domain‐specific ontologies provide definitive frameworks for structuring knowledge, while open‐source software packages enable the creation, validation, manipulation, and sharing of data. To link ontologies with other resources, articles, and multimedia content, a new web‐based platform called the Battery Knowledge Base, which provides a centralized hub to enhance knowledge sharing and collaboration, is introduced. In this article, how these semantic tools address critical challenges in knowledge and data management, driving progress in the field, are highlighted.

## Graphical abstract

Semantic technology bridges the gap between human researchers and artificial intelligence agents, transforming fragmented battery data into interoperable, machine‐readable knowledge. By linking materials, processes, and performance data, it supports advanced discovery and accelerated battery innovation.

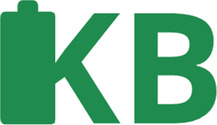

## 1 Introduction

The battery community is generating more data and knowledge than ever before.^[^ ^1^ ^]^ There were more battery research papers published in the 5 years between 2020–2024 than in the entire 33‐year period between 1987–2019.^[^ ^2^ ^]^ If a researcher reads 5 papers per week, it would cover less than 1% of the battery research published in 2024. The volume of battery knowledge being published begins to exceed the capacity of humans to process or even understand it all. At the same time, the tools we employ for documenting and sharing that knowledge have not kept pace with advances in information technology. Scientific knowledge today is still largely documented in freeform text, narrative‐style research papers, and static images intended as print, rather than digital media.^[^ ^3^ ^]^ The knowledge sharing systems of the past simply cannot cope with the massive influx of new data.

Artificial intelligence (AI) tools, like large language models (LLMs), offer one part of the solution to this challenge. Since OpenAI launched ChatGPT in November 2022, the ability to quickly review and summarize information from many different unstructured sources has provided a significant asset for overstretched researchers.^[^ ^4^ ^]^ Although LLMs are able to process unstructured information, they are more efficient, reliable, and correct when fed with structured data. Furthermore, the energy demands from AI queries are placing a significant load on the electricity grid. A recent study found that a query from ChatGPT requires roughly 10 times more energy to process than a Google search.^[^ ^5^ ^]^ The energy consumption of data centers in the United States is projected to consume between 4.6%–9.1% of the total domestic electricity production by 2030.^[^ ^6^ ^]^

Semantic technologies offer a promising solution to these challenges by providing a framework for structuring knowledge and linking data in ways that bridge the gap between human understanding and machine processing.^[^ ^7^, ^8^ ^]^ By enabling data to be encoded in a structured and machine‐readable format, these tools can reduce the computational overhead of unstructured data processing while enhancing accuracy and reliability.^[^ ^9^ ^]^

Semantic technology resources, like ontologies and knowledge bases, are at the core of this transformation.^[^ ^10^ ^]^ These resources provide the structure necessary for effective knowledge sharing, are compatible with the FAIR (Findable, Accessible, Interoperable, and Reusable) principles for scientific data,^[^ ^11^, ^12^ ^]^ and address the inherent complexities of integrating data from diverse sources. As AI becomes increasingly embedded in battery research, the ability to formalize and share knowledge in a machine‐readable way is more critical than ever.

This article examines the role of semantic technology resources in accelerating battery research, with a specific focus on the resources being developed and deployed through the BATTERY 2030+ project. It highlights ontology as a definitive and comprehensive framework for structuring knowledge and introduces a new web‐based platform called the Battery Knowledge Base (BKB), which is a centralized hub for exploring ontologies and linking them together with wiki‐style articles, datasets, and multimedia content in the battery domain. By addressing key challenges in data management and collaboration, we demonstrate how these tools are driving innovation in the field. Finally, we explore the future opportunities and challenges for semantic technologies, emphasizing the importance of open collaboration and community‐driven solutions in shaping the next generation of battery research.

## 2 Semantic Technology

Semantic technology enables both humans and machines to interpret and act on data by structuring knowledge in a meaningful, machine‐readable format. It uses formal standards to convey the meaning of data and information, ensuring interoperability and facilitating advanced automated data processing.

The modern semantic technology stack is built largely on the World Wide Web Consortium (W3C) standards.^[^ ^13^ ^]^ The World Wide Web was initially developed to help researchers at CERN to manage information by linking documents with hypertext.^[^ ^14^ ^]^ However, as the Web expanded, the need for structured data and meaningful relationships became evident, leading to the development of the Semantic Web.^[^ ^15^ ^]^ This extension of the Web created a network of linked data that was annotated with meaningful metadata and designed to be navigated by machine agents, including web crawlers, search engines, and AI tools.^[^ ^16^ ^]^

Semantic technologies are built on a few core principles.^[^ ^7^ ^]^ First, they use persistent unique identifiers to reference resources such as datasets, publications, or equipment, ensuring precise identification and retrieval. Second, they incorporate controlled vocabularies and ontologies, which standardize terminology and formalize the relationships between terms to improve consistency across datasets. Third, they employ machine‐readable formats like JSON–LD, which embed semantic annotations directly within data files.^[^ ^17^ ^]^

The semantic technology stack provides a hierarchical framework for organizing and utilizing structured data. At its foundation, the Resource Description Framework (RDF) expresses information as a network graph comprised of many node‐edge‐node structures called triples.^[^ ^18^ ^]^ A triple is a three‐positional statement that expresses some basic piece of information. For example, we could create a triple to make a statement like, “this article ‐ is published in ‐ *ChemSusChem*”. While this triple is understandable for humans, we need some machine‐readable reference vocabulary to formalize ideas like “is published in” as a standard and shared term. Semantic vocabularies fill this need. Vocabularies define standardized terms, ensuring consistent data interpretation. Ontologies build upon vocabularies by introducing logic and constraints that support reasoning and enable complex queries. Ontologies contain general classes and relationships, but do not reference any specific instances of data; that is the role of a knowledge graph. Knowledge graphs extend ontologies and vocabularies to create networks containing instances of data that are mapped back into the ontology framework.^[^ ^19^, ^20^ ^]^ Finally, knowledge bases serve as repositories for structured data, combining a knowledge graph with wiki‐style articles and multimedia content to make them useful for both humans and machine agents.^[^ ^21^ ^]^ Together, these technologies transform fragmented data into integrated, actionable insights that drive scientific progress.

Semantic technologies are already ubiquitous in the digital world and are foundational to the functioning of the modern Web. Search engines like Google use knowledge graphs to understand relationships between webpages and their content, improving the relevance of the search results.^[^ ^22^ ^]^ Virtual assistants rely on structured ontologies to interpret user queries and provide accurate responses.^[^ ^23^ ^]^ E‐commerce platforms leverage linked data to recommend products based on user behavior, while social media platforms use semantic analysis to organize content.^[^ ^24^, ^25^ ^]^ As the amount of data on the Web continues to increase, it is essential to make it accessible and understandable for machine agents.

Semantic technologies were adopted in scientific research in the late 20th century, with bioinformatics leading the way.^[^ ^26^ ^]^ The Protein Data Bank (PDB) is a key early example. The PDB is a foundational repository for 3D protein structures that enhances data accessibility and integration by incorporating structured metadata and ontologies.^[^ ^27^ ^]^ By encoding the relationships between data elements and ensuring interoperability, the PDB became a valuable resource for researchers worldwide. As the role of AI continues to grow, this resource (and others like it) has facilitated AI‐driven advancements in protein modeling. AlphaFold, which received the Nobel prize in Chemistry in 2024, leveraged PDB's extensive dataset to train its model,^[^ ^28^ ^]^ demonstrating the power of well‐structured semantic data in accelerating discovery. The PDB is not an isolated case; its practices reflect broader trends in applying semantic technologies across scientific domains. Related efforts like the Protein Ontology (PO) and the Gene Ontology (GO) have created an ecosystem of interconnected knowledge that allows deeper insights into biology.^[^ ^29^, ^30^ ^]^

The use of semantic technologies, more broadly in scientific research, has been accelerated by the proliferation of FAIR principles.^[^ ^11^ ^]^ Semantic technologies are one set of tools that can help implement the FAIR principles by structuring data with links to standardized vocabularies,^[^ ^31^, ^32^ ^]^ which is a central building block for accelerated materials research in general.^[^ ^33^ ^]^ Findability is ensured through persistent identifiers and standardized metadata. Accessibility is enhanced via linked data frameworks like RDF and JSON–LD, enabling seamless retrieval. Interoperability relies on ontologies and controlled vocabularies, ensuring semantic consistency across datasets. Reusability is supported by semantic annotations that capture provenance, licensing, and context. As the FAIR principles become more deeply integrated into research funding mandates, researchers are in need of semantic technology resources to help them get the most value from their data.

## 3 Battery Semantic Resources

Semantic technologies in battery research address the challenges of data fragmentation and the increasing complexity of datasets.^[^ ^34^ ^]^ By leveraging structured frameworks that were developed by the Semantic Web and bioinformatics, these technologies support linking data across battery materials, components, processes, and performance metrics.

Pioneering work in bioinformatics demonstrates the immense value of structuring scientific knowledge. These efforts were both ambitious and transformative, creating foundational frameworks for data sharing and analysis that accelerate discovery across the life sciences. However, directly applying these approaches to materials research is less straightforward.^[^ ^35^ ^]^ Battery research spans a far broader and more heterogeneous landscape. It covers materials chemistry, electrochemical processes, multiscale manufacturing, diverse testing protocols, simulation, lifecycle analysis, and more, each with its own specialized terminology, data standards, and methods. This diversity makes it challenging to create a logically consistent semantic framework capable of capturing the full scope of battery research within a single ontology. Addressing this challenge requires a flexible and modular approach capable of linking diverse concepts across the entire battery value chain. The Elementary Multiperspective Materials Ontology (EMMO) provides a top‐ and mid‐level ontology framework specifically designed to express knowledge across materials science domains.^[^ ^36^, ^37^, ^38^ ^]^ EMMO combines philosophical rigor with practical engineering needs, creating a logically consistent foundation for describing materials and their properties. By offering a common semantic layer, EMMO supports interoperability between diverse materials, science domains, and facilitates data integration and reuse across disciplines. Its modular architecture allows domain‐specific extensions, like the Battery Interface Ontology (BattINFO), to inherit this consistent upper‐level framework, while introducing specialized vocabularies and concepts tailored to their particular research needs.

This article focuses mainly on resources related to BattINFO, but there are several other ontology initiatives that address specific challenges across the battery value chain. These efforts highlight how semantic technologies can be applied in a flexible way to address many different challenges in battery research. Ontologies have been used to develop traceability systems that track materials and processes in lithium‐ion battery manufacturing, enabling better inline quality control and process optimization.^[^ ^39^, ^40^ ^]^ In battery testing, hierarchical and interoperable data formats have been designed to describe test results and metadata, ensuring reproducibility and scalable data sharing.^[^ ^41^ ^]^ Other initiatives focus on linking material attributes to production stages to support predictive analytics,^[^ ^42^ ^]^ improving state of charge estimation for electric vehicle battery packs through semantic modeling,^[^ ^43^ ^]^ and integrating ontologies with AI to automate the disassembly of retired batteries.^[^ ^44^ ^]^ Ontology‐driven knowledge graphs have also been introduced to manage and share critical battery data, supporting more efficient data integration and reuse.^[^ ^45^ ^]^ These specialized efforts highlight how flexible, domain‐specific ontologies can complement broader frameworks by addressing targeted needs across the battery domain.

Beyond ontologies, knowledge bases serve as community‐driven platforms for aggregating and disseminating structured information. The BKB, built on OpenSemantic Lab,^[^ ^46^ ^]^ extends BattINFO by serving as a community‐driven platform for structured battery knowledge. The BKB offers a machine‐readable knowledge graph that structures data with semantic concepts from BattINFO, and is supplemented with traditional wiki‐style articles that contain human‐readable multimedia content. Researchers can take advantage of the structured data in the knowledge graph and make contributions to human‐readable article content. This community‐driven approach serves as a centralized repository of knowledge about the battery domain.

The following subsections explore in detail how BattINFO and the BKB provide the foundation for semantic knowledge management in battery research. We examine how these tools facilitate linked data exchange, advanced querying, and supporting the next generation of digital battery research and innovation.

### 3.1 Battery Interface Ontology

BattINFO is a modular, domain‐specific semantic framework that addresses the challenges of managing knowledge across the diverse and rapidly evolving landscape of battery research.^[^ ^34^, ^47^ ^]^ Originally created within the EU project BIG–MAP, BattINFO continues to develop as a resource within BATTERY 2030+ and the global battery community. As part of the EMMO ecosystem, BattINFO leverages EMMO's foundational concepts to ensure semantic consistency with other materials science domains, enabling interoperability across disciplines and multiscale modeling workflows. BattINFO enhances this foundation with specialized, modular vocabularies covering chemical substances, characterization methodologies, and electrochemistry, ensuring that the ontology can evolve alongside advances in the field. This modular design, combined with adherence to EMMO's guiding principles, allows BattINFO to act as a semantic backbone for battery research while also serving as a scalable foundation for more specialized ontologies. This has already been demonstrated in related efforts focused on battery cell manufacturing and battery testing.^[^ ^48^, ^49^ ^]^

The implementation of BattINFO adheres to W3C Semantic Web standards, enabling integration with widely used web‐based knowledge platforms such as Wikidata, DBpedia, and Google's Knowledge Graph. This ensures that battery‐related information is both discoverable and linked to broader scientific and technical datasets. The ontology is published in Turtle (Terse RDF Triple Language) and maintained in public GitHub repositories to support transparent development, version control, and community contributions. Persistent identifiers, managed through the w3id.org PURL service, ensure stable referencing over time even if hosting locations change.

A key feature of BattINFO is its dynamic response adaptation, allowing it to distinguish between human and machine agents. When accessed through a web browser, the ontology redirects users to human‐readable documentation, while machine agents receive machine‐readable RDF representations. To further support its practical use, BattINFO provides pre‐compiled JSON–LD context files, simplifying the creation and integration of linked data across applications. Through its SPARQL endpoint, BattINFO also supports semantic querying, which allows researchers to perform advanced searches that are centered around concepts rather than data filtering or keyword matching.

From a functional perspective, BattINFO can also serve as a semantic backbone for operational data management plans (DMPs), encoding data flows, dependencies, and data handover points in machine‐readable form.^[^ ^50^ ^]^ This enables automatic generation of project‐wide data inventories, supports alignment with FAIR principles, and provides a clear map of how data moves within and across projects, facilitating reuse beyond the initial project context. This approach has been demonstrated in the research project BIG–MAP and shown how it supports efficient data sharing within larger consortia such as those in BATTERY 2030+.^[^ ^50^, ^51^, ^52^ ^]^

#### 3.1.1 Example Use Cases

The versatility of BattINFO's semantic framework can be best appreciated through practical examples. By applying it to real‐world battery data, researchers can transform traditionally fragmented information into structured, machine‐readable knowledge. The following examples highlight how BattINFO enhances data management across electrolyte formulations, battery cell metadata, and performance testing datasets to enable advanced searches and automated comparisons.

#### 3.1.2 Electrolyte Formulations

Electrolyte formulations consist of multiple solvents, salts, and additives, each affecting the battery performance. A typical plain text description takes the form “1 M ${\text{LiPF}}_{6}$ in DMC:EC:EMC 1:1:1 (vol.) + 2 wt% VC.” This recipe can be translated as 1 mole per liter of lithium hexafluorophosphate (${\text{LiPF}}_{6}$), dissolved in a solvent consisting of equal volumes of dimethyl carbonate (DMC), ethylene carbonate (EC), and ethyl methyl carbonate (EMC) with an additive of 2 weight percent of vinylene carbonate (VC). While this notation is familiar to experts, it lacks the precision necessary for systematic comparison and large‐scale analysis. Although the chemical composition of the lithium salt is clearly described, the composition of the carbonate substances relies on non‐canonical abbreviations. Furthermore, mixing quantities like amount concentration, volume ratios, and weight percentages increases the complexity of the recipe. A better approach is to translate this description into a structured semantic model that clearly defines each component within a controlled vocabulary, linking its composition, properties, and functional roles.

**Figure 1:**
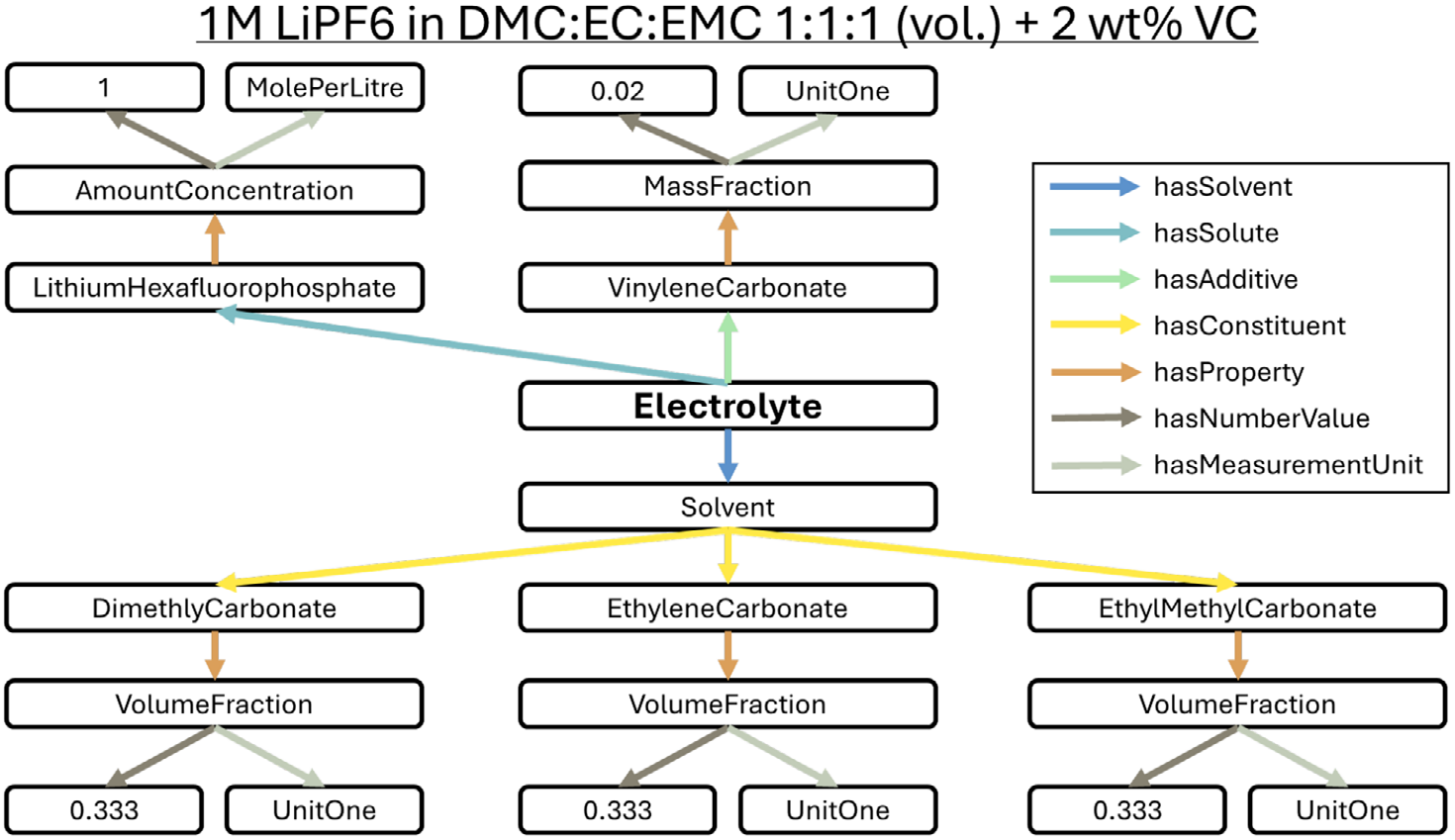
A visual representation of a semantic graph describing the electrolyte formulation “1 M LiPF6 in DMC:EC:EMC 1:1:1 (vol.) + 2 w.t% VC” using ontology terms. Each node is labeled with its skos:prefLabel to support human readability, while the underlying structure is fully machine‐readable through RDF triples. This dual representation facilitates automated querying, data integration, and interoperability. An implementation of this graph in JSON–LD is available in the supplementary materials and on GitHub.^[^ ^53^ ^]^

**Figure** **1** illustrates how ontology‐based modeling transforms textual descriptions into structured, machine‐readable data. For example, the triple [Electrolyte ‐ hasSolute ‐ Lithium Hexafluoro phosphate] establishes that ${\text{LiPF}}_{6}$ is a solute rather than just a listed ingredient. Additional triples, such as [Lithium Hexafluoro phosphate ‐ hasProperty ‐ Amount Concentration], associate a numerical value (1) and a unit (mole per liter), ensuring precise interpretation. As part of EMMO, BattINFO maintains unit consistency by aligning with QUDT, a framework that standardizes physical quantities and measurements in linked data environments.

When the graph is processed by a machine agent, the human‐readable labels are converted to machine‐readable identifiers (e.g., electrolytes). This allows machines to make direct links from BattINFO resources to external resources such as PubChem, Wikidata, Google's Knowledge Graph, and ChEBI, fostering broader knowledge integration. The JSON–LD implementation of the graph and Jupyter Notebook (provided in the supplementary materials and on GitHub^[^ ^53^ ^]^) demonstrate this functionality and show how the query language SPARQL can be used to retrieve information from the graph.

#### 3.1.3 Battery Cell Metadata

Battery performance is influenced by materials, manufacturing parameters, and operational history. Metadata models must comprehensively capture these factors to enable meaningful comparisons and predictive analytics.

Existing metadata descriptions often rely on inconsistent tabular formats or unstructured documentation. The International Electrotechnical Commission (IEC) defines an alphanumeric system for cell identifiers, such as IFpR2032, where “I” denotes a carbon‐based negative electrode, “Fp” indicates an iron‐phosphate positive electrode, and “R2032” specifies a round coin cell format with 20 mm diameter and 3.2 mm height. Although IEC identifiers provide a standardized shorthand for battery cells, they are only superficial descriptions that lack the depth needed for more meaningful understanding. Beyond this basic classification, additional metadata such as nominal voltage, capacity, and manufacturer details are typically stored in separate documents, making integration difficult. To address this challenge, semantic models provide a structured alternative.

**Figure 2:**
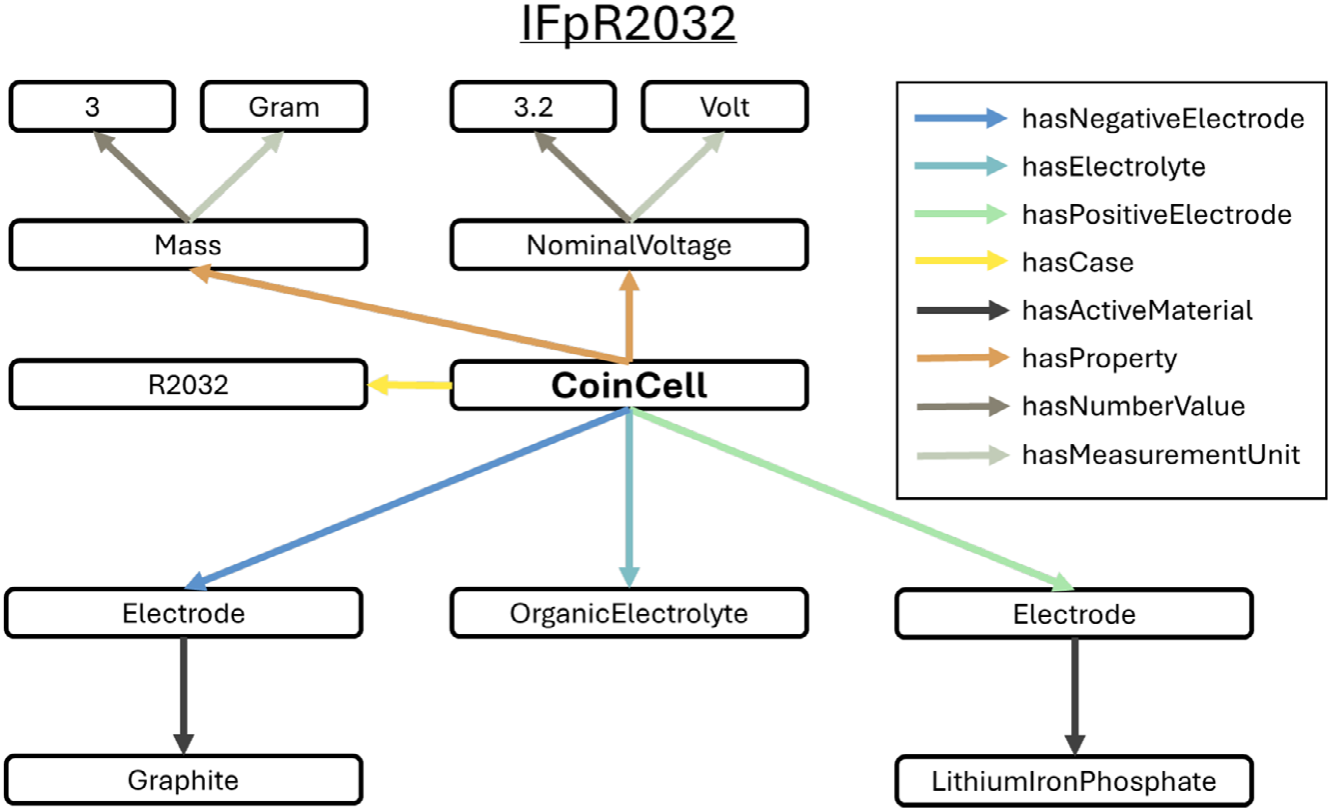
A visual representation of a graph showing the description of an IFpR2032 coin cell. The labels shown in the figure correspond to the skos:prefLabel annotation for the ontology terms. An implementation of this graph in JSON–LD is available in the supplementary materials and on GitHub.^[^ ^53^ ^]^

**Figure** **2** illustrates how an IFpR2032 cell can be represented as a knowledge graph. The alphanumeric code IFpR2032 implies meaning based on assumed pre‐existing knowledge, while the graph makes that meaning explicit. The code letter “Fp,” indicating an iron–phosphate–based electrode is replaced by the triples [CoinCell ‐ hasPositiveElectrode ‐ Electrode] and [Electrode ‐ hasActiveMaterial ‐ LithiumIronPhosphate]. In this way, we can maintain traceability from specific batches of lithium iron phosphate material to specific coin cells. Furthermore, because the BattINFO term for “LithiumIronPhospate” links to other sources like PubChem and Wikidata, agents can follow those links to retrieve additional information (e.g., density and molar mass) that is not explicitly defined in the graph.

This structured metadata representation supports advanced querying so that researchers can filter datasets based on voltage, electrode chemistry, or manufacturing details without manual data processing. These features are demonstrated in the accompanying JSON–LD implementation of the graph and Jupyter Notebook in the supplementary materials and on GitHub.^[^ ^53^ ^]^

#### 3.1.4 Battery Test Metadata

Battery performance testing generates vast time series datasets, containing quantities like voltage, current, temperature, and time. While they are essential for understanding cell behavior, these datasets are often stored in fragmented formats that hinder cross‐study comparisons and automated analyses.

The need for descriptive metadata for tabular datasets extends across many fields, and the CSV on the Web (CSVW) vocabulary has emerged as a widely accepted standard for addressing this challenge. CSVW provides a flexible framework for describing tabular data structures, while BattINFO enriches these descriptions by explicitly linking columns to standardized battery‐related ontology terms, ensuring precise unit definitions and semantic clarity.

**Figure 3:**
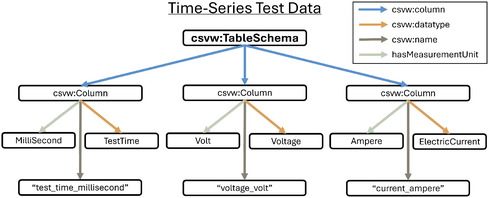
A visual representation of a graph showing the description of a time series test data table schema. The labels shown in the figure correspond to the skos:prefLabel annotation for the ontology terms. An implementation of this graph in JSON–LD is available in the supplementary materials and on GitHub.^[^ ^53^ ^]^

**Figure** **3** visualizes how a battery time series dataset can be mapped to an ontology. By explicitly linking each measurement to an ontology‐defined unit, this approach prevents errors caused by inconsistent unit conventions. Automated validation mechanisms further ensure test condition consistency, improving data reliability. Linking structured metadata to BattINFO terms enables cross‐referencing of battery test data with cell specifications, environmental conditions, and modeling parameters, facilitating holistic performance analysis.

A key advantage of semantic structuring is query‐driven analysis. Instead of manually searching files, researchers can execute SPARQL queries to retrieve specific test conditions, such as identifying all test cycles where voltage drop exceeded a threshold, enabling automated anomaly detection and performance comparisons across different cell designs. The JSON–LD implementation of the graph and Jupyter Notebook (available in the supplementary materials and on GitHub^[^ ^53^ ^]^) demonstrate some of these approaches.

By transitioning from fragmented, text‐based documentation to ontology‐based representations, battery test metadata becomes fully machine‐readable, enabling automated validation, large‐scale integration, and AI‐powered insights into battery performance trends.

#### 3.1.5 Challenges for Broader Adoption

Despite the clear advantages of semantic representation, several technical and adoption challenges must be addressed before these approaches can reach widespread implementation in battery research. Unlike freeform text documentation, structured ontology‐based models require careful metadata annotation and formatting, which can be time‐consuming and technically demanding. While tools and templates can streamline this process, researchers often face a steep learning curve, requiring both training and infrastructure support. The challenge lies in balancing machine‐readability with usability for human researchers unfamiliar with ontological frameworks.

A key challenge for battery ontologies is keeping pace with the continuous growth and evolution of domain knowledge. As new materials, processes, and testing methods emerge, ontologies must be agile enough to incorporate these developments while maintaining internal consistency. Achieving this requires strong community support to ensure that diverse methods and terminology used across different laboratories are adequately represented. At the same time, ontologies must remain a definitive and trustworthy resource that is aligned with established standards. Maintaining and expanding domain ontologies requires active community involvement, an efficient process for creating or modifying terms, continuous updates, and version control to prevent fragmentation.

Many battery datasets are published on platforms like Zenodo, providing valuable open access to research data, but these datasets are often not semantically annotated or linked to relevant metadata, making them difficult to find and reuse effectively. The community needs to do more to encourage and reward the publication of high‐quality, FAIR‐aligned datasets, while also providing researchers with tools and guidance to simplify the process of adding semantic annotations. Developing interoperable data repositories capable of handling heterogeneous data with standardized metadata will be critical to ensuring long‐term value. Domain‐specific infrastructures, such as OPTIMADE for materials design^[^ ^54^, ^55^ ^]^ show how standardized APIs can enhance the discovery and interoperability of materials data within a specific field. More flexible platforms, such as Kadi4Mat,^[^ ^56^ ^]^ offer a broader framework for managing research data across disciplines by combining structured data storage with semantic metadata handling. These efforts also raise the question of how to sustainably fund long‐term storage and curation of curated datasets.

For experimentalists managing large volumes of data under time constraints, manual metadata annotation can present a significant barrier, even when the benefits of semantic technologies are well understood. To overcome this, semantic resources must be supported by intuitive, accessible interfaces and automation tools that streamline the annotation process. For example, the BattINFO converter enables researchers to use familiar spreadsheet templates to generate machine‐readable JSON–LD metadata, eliminating the need to work directly with RDF syntax or ontological files.^[^ ^57^ ^]^ AI‐assisted annotation tools, lightweight web‐based laboratory notebooks, and standardized templates can further reduce the technical burden.

In parallel, semantic validation methods play a critical role in ensuring data integrity and reducing human error. These include syntactic checks (e.g., validating structure against a JSON schema), semantic consistency checks (e.g., flagging mismatches such as a length being annotated with a unit of mass), and ontology‐aware reasoning using inference engines. Frameworks like SHACL, OWL reasoning, or even schema‐based tools such as Pydantic can help catch inconsistencies early in the workflow and guide users to correct them. Community‐curated examples, detailed documentation, and shared best practices also support reproducible use. Moving forward, the development of user‐friendly, AI‐supported interfaces, combined with robust semantic validation, will be essential for scaling adoption across the battery research community.

Addressing these challenges requires collaboration between battery researchers, ontologists, data scientists, and industry stakeholders. Overcoming these barriers will not only enable more efficient data exchange and deeper insights, but also create opportunities for new tools and platforms to streamline adoption. The next section explores how the BKB is working to bridge these gaps and enhance the accessibility and usability of semantic technologies within battery research.

### 3.2 The Battery Knowledge Base

The BKB is a community‐driven, open‐access resource designed to facilitate the structured sharing, management, and retrieval of battery‐related knowledge across the entire value chain. Its scope is intentionally broad. In addition to describing materials, components, cells, and processes, the BKB includes knowledge entries intended to raise the shared baseline of battery understanding, ranging from introductory tutorials to expert commentary and best practices. It also indexes raw datasets and metadata to help users discover relevant information and understand parameter interrelationships. To foster collaboration and awareness of current activities, the BKB supports entries for people, projects, organizations, and events, helping connect the research community through structured semantic information.

Built on Semantic MediaWiki^[^ ^58^ ^]^ and Open Semantic Lab,^[^ ^46^ ^]^ the BKB provides a familiar, wiki‐based interface which combines the concept of Wikipedia and Wikidata,^[^ ^59^ ^]^ enabling researchers, engineers, and industry professionals to collaboratively develop and refine structured battery knowledge. All resources are modeled with ontology‐annotated and object‐oriented linked data schemas (OO–LD)^[^ ^60^ ^]^ which are also available at GitHub.^[^ ^61^ ^]^ Identity is managed by login via ORCID's^[^ ^62^ ^]^ Single Sign‐On (SSO), associating the contributions of users to the corresponding ORCID ID.

The BKB is designed to scale to large volumes of metadata. Since it stores structured semantic metadata and links to external repositories rather than ingesting raw data directly, it can accommodate thousands of dataset records efficiently. The underlying Semantic MediaWiki and Open Semantic Lab infrastructure have been validated in other large‐scale projects, and the BKB leverages these proven technologies to support high‐volume use cases such as battery cycling test indexing.

**Figure 4:**
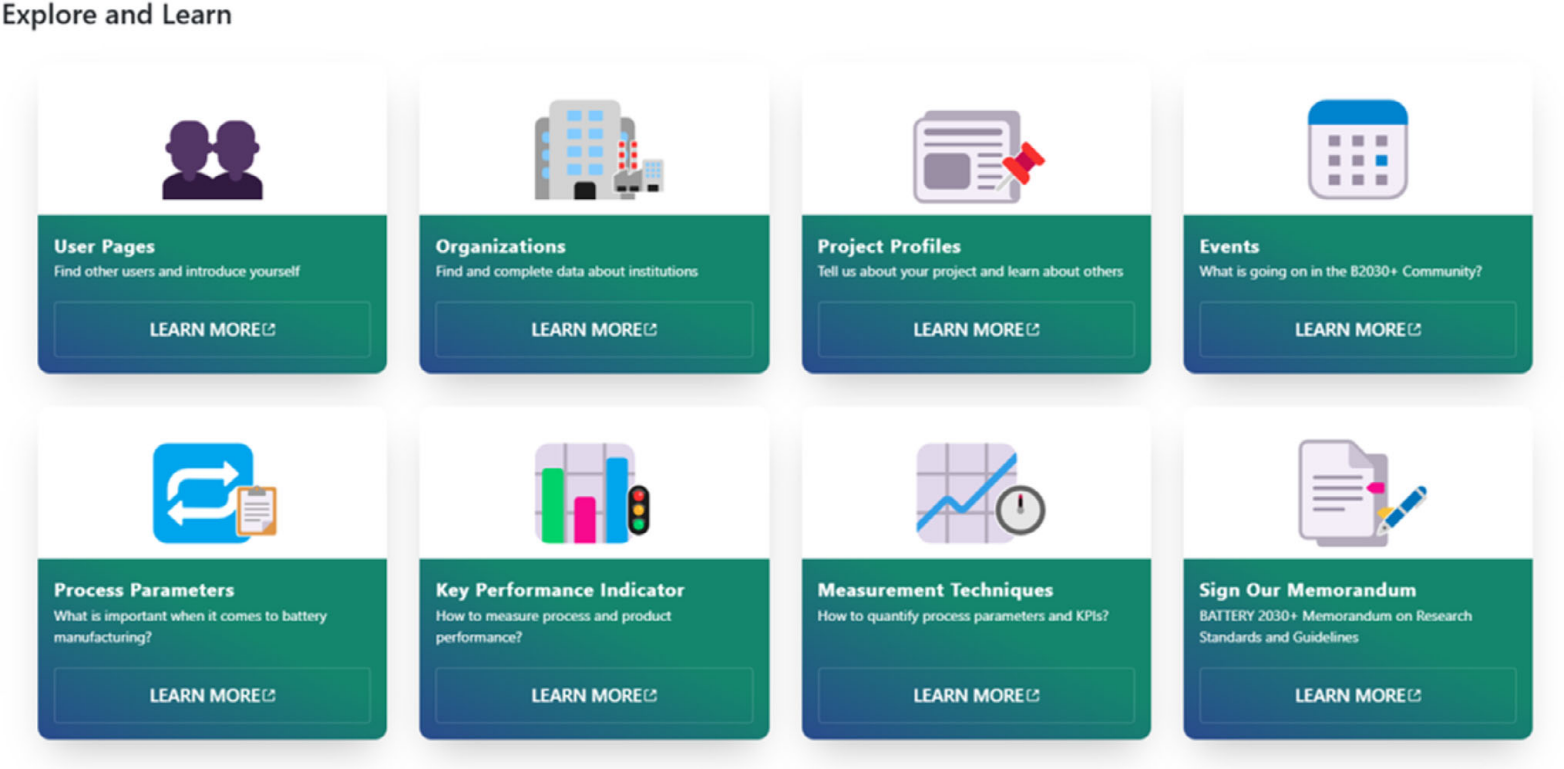
A screenshot of the BKB homepage showing some of the key topics that users can explore. Each topic is represented both as a human‐readable article and as a semantically structured entity in the underlying knowledge graph, enabling both intuitive browsing and machine‐driven querying.

The BKB encompasses a wide range of knowledge categories, making it a versatile resource for different stakeholders. The homepage provides an overview of the core topics that users can explore, which is shown in **Figure** **4**. Like a traditional knowledge base, it includes entries on people, organizations, projects, and events, but it extends far beyond these general topics to incorporate battery‐specific content. Including a full import of the BattINFO and EMMO ontology, the platform ensures that users are able to easily contribute structured information on a diverse array of battery‐related topics, including materials, components, cells, manufacturing, and testing methodologies.

Researchers can engage with the BKB in multiple ways. They can create and edit articles associated with ontology‐defined terms, ensuring that content remains comprehensive and up to date. Researchers can annotate and enrich datasets with metadata, facilitating automated search and retrieval across various studies. Additionally, researchers can create personal pages to highlight their technical expertise, experience, and publications, while project managers can develop pages for their initiatives to share results and consortium information. Event organizers can also use the platform to promote their events and document the outcomes. The open‐access nature of the platform fosters continuous community curation, ensuring that the knowledge base evolves alongside advances in battery science, manufacturing, and performance assessment. The BKB and BattINFO are currently governed by the Battery2030+ consortium, and will transition to a community‐driven governance model after the end of the project.

Beyond human readability, the BKB leverages a semantic architecture that structures information as a knowledge graph. Unlike conventional wikis, where data is stored as unstructured text, this approach enables dynamic querying, filtering, and contextualized knowledge discovery. Instead of relying on simple keyword searches, users can explore battery‐related concepts through meaningful semantic relationships, allowing for deeper insights and more efficient data retrieval. This structured framework also makes the BKB highly compatible with AI‐driven tools, robotic R&D platforms, and predictive modeling software, bridging the gap between human and machine‐readable knowledge.

One of the first practical use case for the BKB was to describe the manufacturing of lithium‐ion batteries, which is a very complex process chain featuring many steps. Each step has many control parameters that, together with the properties of the input materials, affect the quality of the product. Obtaining a thorough understanding of this process chain and its interconnections is essential for both researcher and industry stakeholders. It helps researchers to improve the reproducibility of their results and industrial stakeholders to fine‐tune the quality of their manufacturing process.

**Figure 5:**
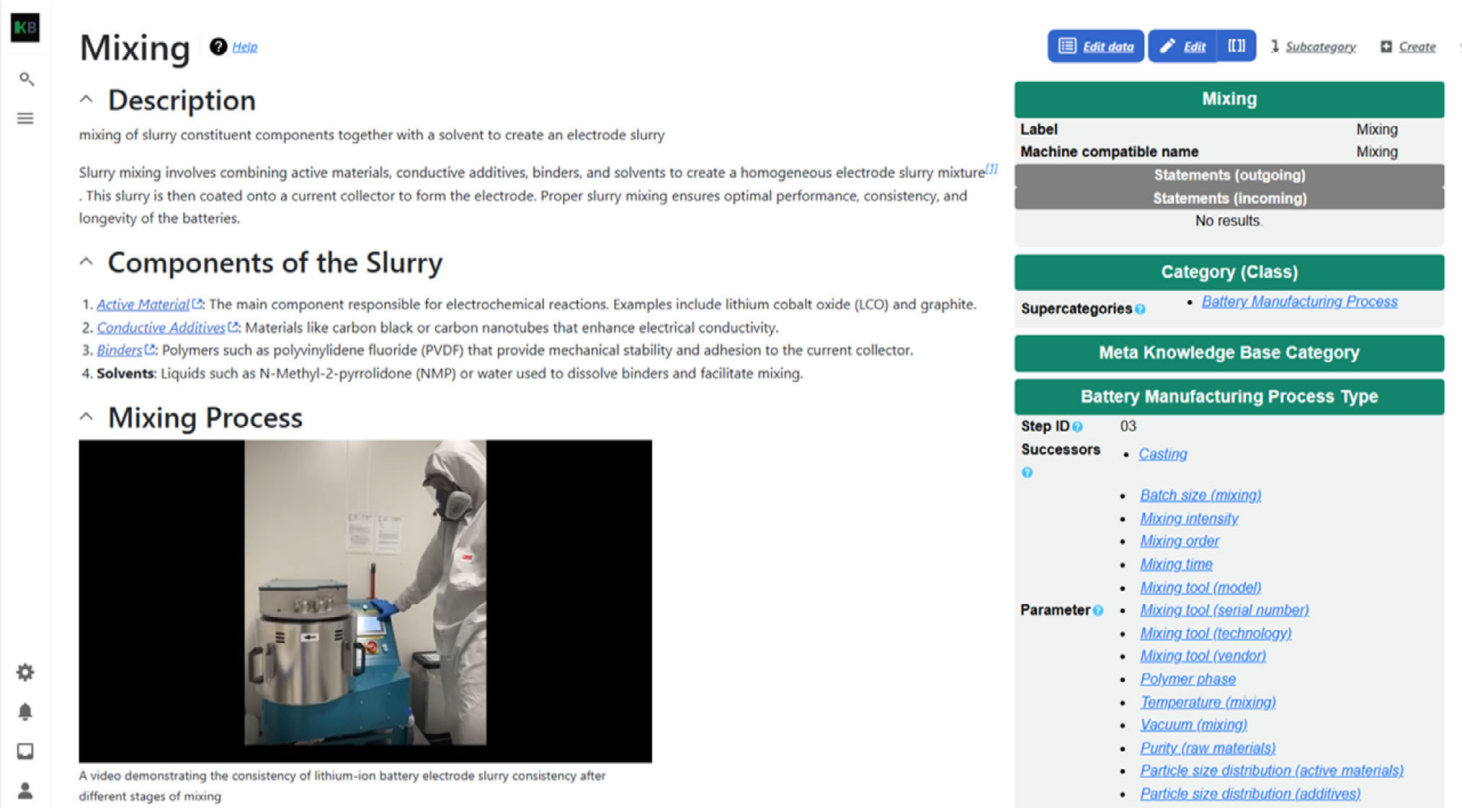
A screen shot of an entry in the BKB describing the process of mixing an electrode slurry. The page integrates a human‐readable wiki article with a semantic knowledge panel (right) that summarizes the structured metadata and ontological relationships. This combination enables both intuitive understanding and machine readability, supporting automation and knowledge retrieval. The article also supports multimedia content such as the embedded instructional YouTube video shown here.

To demonstrate an example of this, **Figure** **5** shows the dedicated page describing the “mixing” process in the lithium‐ion battery manufacturing chain. The page is structured using semantic technologies to integrate both human‐readable explanations and machine‐readable metadata. This page supports human readability with a knowledge panel that summarizes key metadata about the process, including relevant materials, equipment, and procedural details. The page also allows for rich content contributions. Researchers and industry experts can expand the wiki article with detailed explanations, best practices, and citations, ensuring the resource remains comprehensive and up to date. Multimedia content can also be embedded, as demonstrated by an instructional YouTube video on the page, that visually walks through the process of preparing an electrode slurry for lithium‐ion battery production.

**Figure 6:**
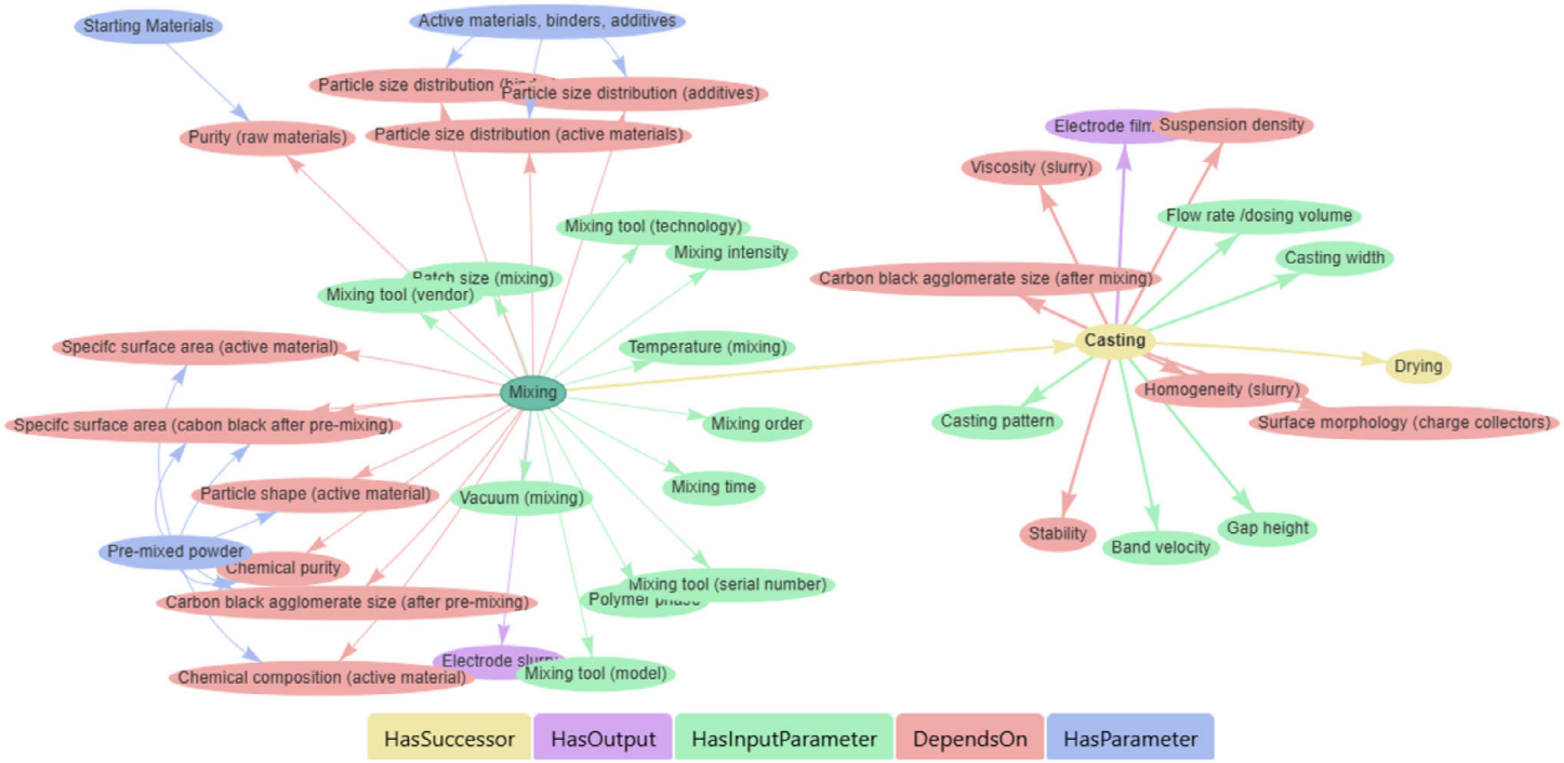
A screenshot of the process graph for the “mixing” step in lithium‐ion battery manufacturing, as represented in the BKB. This machine‐readable graph structures knowledge about the process, including input parameters (green), quality‐relevant quantities (red) and their relationships to materials (blue), outputs (purple), and links to subsequent process steps (yellow). This structured representation connects to the human‐readable article, enabling seamless integration between visual explanations and computational analysis.

Behind the human‐readable content, the BKB leverages the BattINFO ontological framework to structure a machine‐readable knowledge graph describing the process. **Figure** **6** shows a screenshot from the BKB, isolating the part of the knowledge graph related to the mixing process. Each node in the graph represents a concept with its own persistent and unique identifier, which can be annotated with human‐readable information in its own wiki page. The edges connecting the nodes are object properties from the knowledge base that serve to impose structured relationships between the concepts. This approach allows the BKB to express knowledge about the mixing process including its input parameters (green), the key properties (red) of the component materials (blue) that affect the quality of the output product (purple), and links to the next steps in the process chain (yellow). This approach allows machines to understand the context of data that are mapped to these nodes and infer information about how they are related. This combination of structured and semantically rich knowledge, with explanatory text and multimedia makes the BKB an intuitive, yet powerful resource for both human users and machine agents.

The structured, community‐driven nature of the BKB also plays an important role in formalizing standards, protocols, and best practices that enhance the reproducibility and efficiency of battery research. The adoption of common vocabularies and ontologies promotes consistency in terminology and reduces ambiguity in procedural descriptions. Formalizing process descriptions, like the above example, provides clear guidance for researchers and encourages them to follow and report well‐documented, reusable protocols. Over time, as these protocols and recommendations gain wider acceptance, they can shape evolving best practices that are continuously documented and refined in the knowledge base. The collaborative editing and review features of the BKB ensure that the community can collectively improve and update these protocols to reflect the latest scientific understanding and align with recommendations from regulatory bodies, funding agencies, and industry groups.

Another important feature of the BKB is its ability to serve as a repository for structured dataset indexing. Researchers and institutions can contribute datasets to the BKB by uploading them on Zenodo in the BKB community.^[^ ^63^ ^]^ This community serves as a trusted and curated source of data, which is regularly scrapped for metadata to instantiate entries in the BKB. This enables simplified data sharing while preserving proper attribution and facilitating integration with other research efforts. The BKB links datasets to ontology terms and provides a contextualized approach to battery research, making it easier to find and analyze relevant information.

In addition to the web browser interface, the BKB provides programmatic access through RESTful APIs, a SPARQL endpoint, and a Python package.^[^ ^60^ ^]^ These capabilities allow external applications to retrieve structured battery knowledge, enabling AI‐driven material discovery, predictive modeling, and automated experimental design. For example, researchers can use a SPARQL query to extract all datasets related to lithium‐ion battery degradation under specific cycling conditions, enabling automated insights and large‐scale comparisons. This can also be done by using the Sparklis‐UI to build queries from natural language like, “give me every Dataset that has a relation to LithiumIonBattery.” In addition, the graph is indexed in a RAG approach to enable direct question–answering with LLMs. As an example, users could submit the prompt, “dataset about lithium ion battery” in the search bar to get an answer with source links from the chatbot.

One of the persistent challenges in battery research is the fragmentation of data across different sectors and research environments. The BKB promotes semantic data standards that cover the entire battery value chain, ensuring that data from different aspects are consistently structured and interoperable. By centralizing battery knowledge within a unified framework, the BKB enables researchers to compare experimental results and facilitate cross‐sector collaboration. The use of linked data principles ensures that the BKB's structured knowledge can interoperate with other scientific databases, knowledge graphs, and industrial data infrastructures, bridging the gap between academic research and large‐scale industrial applications.

The BKB advances the state of semantic data management for battery research. By leveraging the capabilities of Semantic MediaWiki and Open Semantic Lab, providing structured knowledge through APIs and SPARQL endpoints, and fostering a community‐driven approach to knowledge curation, the BKB addresses critical challenges in knowledge managment and data standardization. Through its structured, interoperable framework, the BKB facilitates collaboration, enhances data reuse, and accelerates advancements in battery science and technology. More details can be found at the BKB.^[^ ^64^ ^]^

## 4 Summary and Outlook

The increasing volume of battery research and data presents both a challenge and an opportunity for the scientific community. Traditional methods of documenting and sharing knowledge—freeform text in research papers and unstructured datasets—are struggling to keep pace with the exponential growth of new information. At the same time, structured and machine‐readable data formats facilitate AI‐ and machine‐driven research. Semantic technologies, including ontologies and knowledge bases, offer a solution by providing standardized frameworks for structuring and linking battery‐related knowledge.

Semantic resources, like BattINFO and the BKB, can facilitate knowledge integration, improve data accessibility, and enhance interoperability in battery science. BattINFO, a modular ontology framework built within the EMMO, provides a structured vocabulary for battery‐related concepts, enabling precise data representation and linking across research disciplines. The BKB, built on Semantic MediaWiki and Open Semantic Lab, extends these capabilities by offering a community‐driven platform, where researchers can contribute, retrieve, and integrate structured knowledge in a format accessible to both humans and machines. Together, these tools create a foundation for linked data exchange, advanced querying, and AI‐driven insights in battery research.

Through concrete use cases, we have demonstrated the power of structured data in electrolyte formulations, battery cell metadata, and performance test datasets. By transforming unstructured text‐based information into knowledge graphs, researchers can automate data retrieval, perform similarity analyses, and enhance machine learning applications. The integration of SPARQL endpoints, JSON–LD representations, and RDF‐based ontologies supports collaboration between human researchers and machine‐driven analytics, paving the way for AI‐assisted discovery in battery science.

Despite these advancements, several challenges remain in achieving widespread adoption of semantic technologies. More efficient tools are needed to support efficient metadata annotation, and ontology evolution need continued community engagement. Encouraging open data practices and fostering collaboration between research institutions, industry stakeholders, and ontology developers will be essential to overcome these barriers.

Looking ahead, semantic technologies are poised to become an integral part of the digital battery ecosystem. Future developments should focus on expanding ontology coverage, improving automated knowledge extraction methods, and developing intuitive interfaces for researchers to engage with structured data more easily. As funding agencies and regulatory bodies increasingly emphasize FAIR principles, researchers will need robust semantic frameworks to maximize the impact and usability of their data.

Better structuring of knowledge can reduce duplication of effort and ensure that valuable resources such as materials, energy, funding, and time are used more efficiently. By enabling broader reuse and integration of datasets, semantic tools accelerate the translation of research findings into industrial applications. This not only supports faster innovation cycles but also contributes to more sustainable battery development, reducing environmental impact and promoting responsible use of public and private research investments for societal benefit.

By continuing to refine and expand these tools, the battery research community can accelerate scientific discovery and enable the next generation of data‐driven battery innovations. The BKB and BattINFO represent important first steps in this transformation, but their long‐term success will depend on ongoing contributions from the global research community.

## Conflict of Interest

The authors declare no conflict of interest.
